# Characterization of Hormonal, Metabolic, and Inflammatory Responses in CrossFit® Training: A Systematic Review

**DOI:** 10.3389/fphys.2020.01001

**Published:** 2020-08-28

**Authors:** Nacipe Jacob, Jefferson S. Novaes, David G. Behm, João G. Vieira, Marcelo R. Dias, Jeferson M. Vianna

**Affiliations:** ^1^Medical and Health Sciences College of Juiz de Fora - Suprema, Juiz de Fora, Brazil; ^2^Postgraduate Program in Physical Education of Federal University of Juiz de Fora, Juiz de Fora, Brazil; ^3^Department of Gymnastics, Federal University of Rio de Janeiro, Rio de Janeiro, Brazil; ^4^School of Human Kinetics and Recreation, Memorial University of Newfoundland, St. John's, NL, Canada; ^5^Laboratory of Exercise Physiology and Morphofunctional Assessment of Granbery Methodist College, Juiz de Fora, Brazil

**Keywords:** CrossFit, high-intensity functional training, metabolism, physical functional performance, physiology

## Abstract

**Background:** CrossFit® training is a high-intensity functional training program that aims to increase physical functional performance through biochemical responses, i.e., hormonal, metabolic, and inflammatory responses. Most hormonal, metabolic, and inflammatory changes induced by CrossFit® training have been reported in isolated clinical studies. The purpose of this review was to systematically explore the existing literature on characterization of hormonal, metabolic, and inflammatory responses resulting from CrossFit® training.

**Methods:** A systematic search of the literature was conducted in PubMed, Web of Science and Scopus from August 2019 to October 2019. Studies were selected through critical review of the content. Using specific keywords, 623 articles were found, of which 597 were excluded for ineligibility, and 25 were eligible. The papers were separated according to subject area: hormonal (*n* = 8), metabolic (*n* = 19), and inflammatory (*n* = 6) changes. All were published between 2015 and 2019.

**Results:** This review reveals potential effects of CrossFit® training on hormonal, metabolic, and inflammatory responses. However, studies had low levels of evidence and reliability due to methodological limitations.

**Conclusion:** In summary, the results showed a greater volume and intensity of workouts accentuate the responses, that are of paramount importance for improving understanding of the effects of CrossFit^®^ training and serve as a basis for prescribing future exercise protocols.

## Introduction

High-intensity functional training (HIFT) is a training program that emphasizes functional movements. HIFT uses a combination of movements, and self-selected time periods of work and rest (Feito et al., 2018). An example of HIFT is CrossFit^®^ training, that is a constantly varied and high-intensity functional training program. The training consists of a combination of different exercise elements: cardiovascular (CV), gymnastic and weightlifting exercises (Glassman, 2017). The combination and variation of these different elements promotes an improvement in fitness. CrossFit^®^ training session, i.e., the workout of the day (WOD), is structured into joint mobility, warm-up, a technical portion, and the main portion. WODs are performed with short or no breaks between exercises, repetitions, and rounds (Glassman, 2007). Usually, WODs are designed to perform the exercise in the shortest possible duration or to perform as many repetitions or rounds as possible (AMRAP) in a given period of time.

Exercise works as a stressor during and after its execution and can cause inflammation (Silveira et al., 2016). These responses, which can be induced by stressors and are vital for host defense and natural tissue homeostasis, initiate the elimination of noxious compounds and damaged tissue (Allen et al., 2015). Apparently, several hormonal (Kliszczewicz et al., 2018a; Mangine et al., 2018; Cadegiani et al., 2019), metabolic (Maté-Muñoz et al., 2018; Tibana et al., 2018; Feito et al., 2019a) and inflammatory (Michnik et al., 2017; Durkalec-Michalski et al., 2018) changes occur with CrossFit^®^ practice. Emphasizing the strong relationship between the hormonal, metabolic, and inflammatory profiles that occur with systematic practice of this high-intensity activity is important (Coco et al., 2019; Tibana et al., 2019b). Thus, an understanding of the hormonal, metabolic, and inflammatory molecular changes is fundamental to establishing the response parameters that come with training.

Studies on the physiological changes resulting from CrossFit^®^ training have been published, but they exhibit large methodological discrepancies, which presents difficulties in explaining the results. The studies present protocols with different time lengths (3.5–23.3 min), exercise modalities (CV, gymnastic, and/or weightlifting exercises), methods (for time or AMRAP), and intensities (absolute or relative load). In reviewing the literature, it was observed that most of the hormonal, metabolic, and inflammatory changes related to CrossFit^®^ training have been reported in isolated clinical studies.

To date, no systematic review of such changes has been performed. The purpose of this review was to systematically review the existing literature on characterization of hormonal (testosterone, cortisol, growth hormone (GH), insulin-like growth factor 1 (IGF-1), adrenaline, noradrenaline, metabolic [lactate, glycemia, cholesterol, creatinine, glutamic oxaloacetic transaminase (GOT), glutamic pyruvic transaminase (GPT)], and inflammatory [interleukin (IL)-6, IL-10, creatine kinase (CK)] responses associated with CrossFit^®^ training.

## Methods

A systematic literature search was conducted in accordance with the Preferred Reporting Items for Systematic Reviews and Meta-Analyses (PRISMA) guidelines (Liberati et al., 2009).

### Eligibility Criteria

This research followed the PICOS strategy to develop the search criteria and determine which relevant articles to include or exclude.

1. Participants: Men and women over 18 years old.
2. Interventions: Any type of intervention that evaluates hormonal, metabolic and inflammatory changes that occur after training protocols based on CrossFit^®^ training.
3. Comparators: The CrossFit^®^ training protocols were compared, provided that there were different training methods.
4. Outcomes: The results reported changes related to hormonal (testosterone, cortisol, GH, IGF-1, adrenaline, and noradrenaline), metabolic (glycemia, cholesterol, creatinine, GOT, and GPT) and inflammatory (IL-6 and IL-10) parameters. CK, that is biomarkers of muscle damage, was included in inflammatory responses.
5. Study design: Randomized and non-randomized trials, using either cross-over or parallel groups, comparing different types of CrossFit^®^ training interventions.

### Selection Criteria

The specific inclusion criteria were as follows: (1) articles that were original research; (2) intervention based on CrossFit^®^ training; (3) a sample of men and women; (4) studies that investigated at least one hormonal, metabolic, or inflammatory/muscle damage variable relevant to the analysis in the present study. Studies were excluded in the following cases: (1) duplicate articles; (2) articles that were not in the English language; (3) articles that presented training protocols not based on CrossFit^®^ training; (4) articles with special populations; (5) articles that were systematic reviews, conference abstracts, dissertations, theses, and book chapters.

### Search Methods for Identification of Studies

The systematic literature search was carried out until October 2019 using the following databases: PubMed, Web of Science and Scopus. The articles were searched using a combination of keywords corresponding to the theme of the review: CrossFit OR “high-intensity functional training” OR HIFT. Medical Subject Headings (MeSH) was consulted to check possible entry terms related to the keywords. After combining the research results and discarding duplicate studies in the databases, two researchers (NJ and MRD) independently selected titles and abstracts to identify relevant studies. The included articles were retrieved, read in full (full text) and independently assessed for eligibility by the same two researchers (NJ and MRD) according to the criteria described above. A meeting was held, and in the case of disagreement regarding the selection of articles, a third author (JN) was consulted to resolve the disagreement.

### Data Collection and Analysis

**Figure 1:**
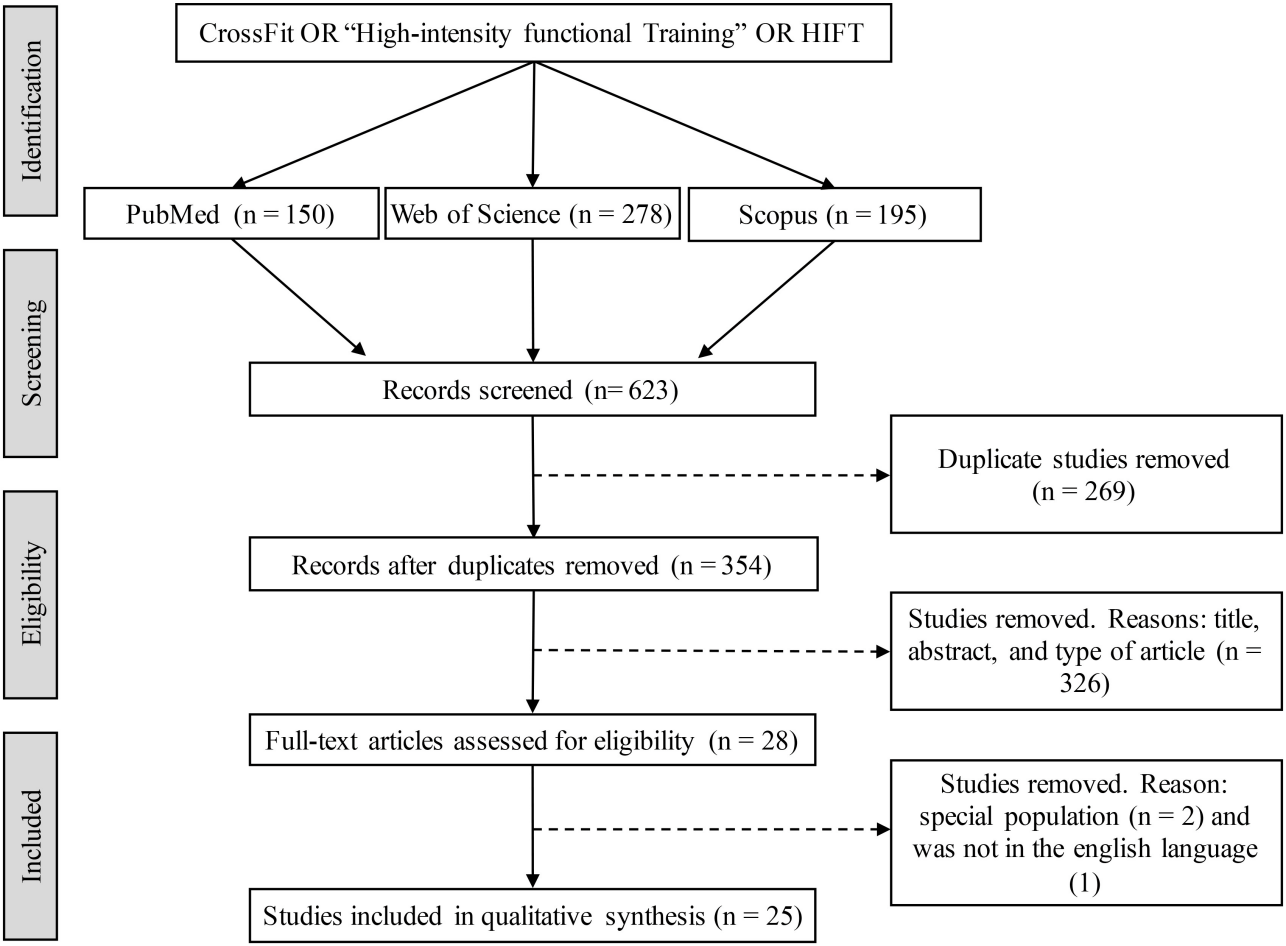
Flow diagram of the study selection process.

Standardized data extraction forms were completed by two researchers (NJ and MRD) and verified by another researcher (JN). Information on the type of study design, characteristics of the participants, sample size, time of experience in the profile, data collection, CrossFit^®^ training protocols, and main conclusions was extracted (see Tables 1–3). The entire study selection process is shown in Figure 1.

**Table 1:** Hormonal responses associated with CrossFit^®^ training.

**References**	**Experimental design**	**Sample (*n*); profile experience time**	**Data collected**	**Training protocol**	**Main findings**
Kliszczewicz et al. (2017)	Acute	Men (*n* = 10); trained ≥3 months	Pre, post, and 1, 3, and 6 h post	WOD 1—For time: 30 power C&J WOD 2−15′ AMRAP: 250 m row, 20 KB swings, 15 dumbbell thrusters	Ad and NA: ↑ post Ad: ↓ 3 h post
Kliszczewicz et al. (2018a)	Acute	Men (*n* = 10); trained ≥3 months	Pre, post, and 1, 3, and 6 h post	WOD 1—For time: 30 power C&J WOD 2−15′ AMRAP: 250 m row, 20 KB swings, 15 dumbbell thrusters	IGF1: NS GH: ↑ post and 1 h post-WOD 2
Kliszczewicz et al. (2018b)	Acute	Men (*n* = 10); trained ≥3 months	Pre, post, and 1, 3, and 6 h post	WOD 1—For time: 30 power C&J WOD 2−15′ AMRAP: 250 m row, 20 KB swings, 15 dumbbell thrusters	Ad and NA: ↑ post
Mangine et al. (2018)	Acute	Men (*n* = 5) and women (*n* = 5); trained >2 years	Pre, post, and 30′ and 60′ post	Weeks 1−20′ AMRAP: 25 ft overhead walking lunge, 8 burpees over the bar Weeks 2–5 ×4′ AMRAP: 25 toes to bar, 50 DU and 15-13-11-9-7 squat cleans Weeks 3−7′ AMRAP: 10 power snatches and 3 bar muscle-ups Week 4−13′ AMRAP: 55 deadlifts, 55 wall balls, 55 cal rows and 55 HSPUs Week 5—Time to conclusion: 21-18-15-12-9-6-3 de thrusters and burpees over the bar	T: ↑ post in weeks 2–5 (in relation to pre) and ↑ 30′ and 60′ post in weeks 3 and 5 (in relation to pre) C: ↑ post and 30′ post in weeks 1–5 and ↑ 60′ post in weeks 1 and 5
Cadegiani et al. (2019)	Descriptive of cross-sectional cohort	Healthy men (*n* = 21) and men with overtraining (*n* = 9); trained ≥6 months; inactive men (*n* = 10)	6 months post	Training protocol not described	T: > Healthy men (in relation to men with overtraining) GH: > Healthy men (in relation to inactive men) C rest, IGF-1, Ad: NS NA: > men with overtraining
Mangine et al. (2019)	Acute	Men (*n* = 5) and women (*n* = 5); trained >2 years	Pre, post, and 30′ and 60′ post	WOD 1: 7′ AMRAP: 10 power snatches and 3 bar muscle-ups WOD 2: 13′ AMRAP: 55 deadlifts, 55 wall balls, 55 cal rows and 55 HSPUs	Ad: ↑ post-WOD 1 and 2 NA: ↑ post-WOD 2 and ↓ 60′ post-WOD 2 (in relation to pre)
Poderoso et al. (2019)	Longitudinal	Men (*n* = 17) and women (*n* = 12); trained >6 months	Pre and 2°, 4°, and 6° months post	6 months of CrossFit^®^ training not described for 5 days per week	T: ↑ 6 months (NS in women) C: ↓ 4 months (in relation to pre) and ↓ 6 months (NS in women)
Tibana et al. (2019a)	Acute	Men (*n* = 9); trained >6 months	24 h pre and 24, 48, and 72 h postcompetition	WOD 1—For time in group *Athlete A*: Row 500 m, 30 strict HSPUs, 15 ring muscle-ups *Athlete B*: Row 1,000 m, 24 strict HSPUs, 12 ring muscle-ups *Athlete C*: Row 1,500 m, 18 strict HSPUs, 9 ring muscle-ups WOD 2—For time for each athlete: 15 fat bar hang power cleans (40 kg), 20 m overhead walking lunges (40 kg), 25 toes to bars, 10 fat bar shoulder to overhead (40 kg), 20 m overhead walking lunges (40 kg) WOD 3—For time for all athletes: 27 burpees box jump-over, 21 legless rope climbs WOD 4—For time for each athlete: 15 m handstand walk, 6-4-2 squat snatches (60-70-85 kg), 15 m handstand walk WOD 5—For time for all athletes: 30 cal assault bike, 20 thrusters (50 kg), 40 cal assault bike, 16 thrusters (60 kg), 50 cal assault bike, 12 thrusters (70 kg), 60 cal assault bike, 8 thrusters (75 kg)	T and C: ↓ until 48 h post

*WOD, workout of the day; C&J, clean and jerk; AMRAP, as many rounds/repetitions as possible; KB, kettlebell; Ad, adrenaline; NorAdr, noradrenaline; IGF-1, insulin-like growth factor 1; GH, growth hormone; DU, double under; HSPU, handstand push-up; T, testosterone; C, cortisol; NS, not significant*.

**Table 2:** Metabolic responses associated with CrossFit^®^ training.

**References**	**Experimental design**	**Sample (*n*); profile experience time**	**Data collect**	**Protocol of training**	**Main finding**
Fernandez-Fernandez et al. (2015)	Acute	Gender not described (*n* = 10); trained >12 months	Pre, post, and 1′, 3′, and 5′ post	WOD 1 (Fran)−3 RFT: 21-15-9 thrusters and pull-ups WOD 2 (Cindy)−20′ AMRAP: 5 pull-ups, 10 push-up, and 15 squats	Lactate: ↑ post in both WODs (non-significant differences between WODs)
Murawska-Cialowicz et al. (2015)	Longitudinal	Men (*n* = 7) and women (*n* = 5); trained >3 months	Pre and 3 months post	3 months of CrossFit^®^ training not described for 2 days per week	Lactate: NS
Shaw et al. (2015)	Acute	Men (*n* = 12); inactive >6 months	Pre and post	10′ AMRAP: 3 burpees, 4 push-ups, and 5 squats	Lactate: ↑ post Glycemia and cholesterol: NS
Escobar et al. (2016)	Acute	Control (*n* = 9) and carbohydrate suppl. (*n* = 9) groups with men (*n* = 7) and women (*n* = 11); trained >12 months	Pre, 4′ and 8′ (during), and post	12′ AMRAP: 20 box jump, 6 KB thrusters, and 6 bar-facing burpees	Lactate: ↑ 4′, 8′, and post in both groups, with difference between groups NS
Perciavalle et al. (2016)	Acute	Men (*n* = 15); athletes of CrossFit^®^ training	Pre, post, and 15′ post	For time: 27–21–15–9 cal row and thrusters (43 kg)	Lactate: ↑ post Glycemia: NS
Tibana et al. (2016)	Acute	Men (*n* = 9); trained >6 months	Pre and post	WOD 1−5 ×1 snatch block (2–5′ rest); 3 × 5 snatches full (90″ rest); 3 × 60″ Plank hold (90″ rest); 10′ AMRAP: 30 DUs, 15 power snatches WOD 2−5 × 1 clean blocks (2′ rest); 5 × 1 jerks (2′ rest); 3 × 5 clean fulls (90″ rest); 3 × 10 strict HSPUs (2′ rest); 12′ AMRAP: 250 m row, 25 burpees touch target	Lactate and glycemia: ↑ post in both WODs and > after WOD 1 than after WOD 2
Escobar et al. (2017)	Acute	Men (*n* = 7) and women (*n* = 11); trained >12 months	Pre, 4′ and 8′ (during), and post	12′ AMRAP: 20 box jumps, 6 KB thrusters, and 6 bar-facing burpees	Lactate: ↑ 4′, 8′, and post
Kliszczewicz et al. (2017)	Acute	Men (*n* = 10); trained ≥3 months	Pre, post, and 1, 3, and 6 h post	WOD 1—For time: 30 power C&Js WOD 2−15′ AMRAP: 250 m row, 20 KB swings, 15 DB thrusters	Glycemia: ↑ post for both WODs
Maté-Muñoz et al. (2017)	Acute	Men (*n* = 34); trained >6 months in strength training	Pre and 3′ post	WOD 1 (Cindy)−20′ AMRAP: 5 pull-ups, 10 push-ups, and 15 squats. WOD 2 (Tabata)−8 × 20″/10″ rest DUs WOD 3−5′ AMRAP: power cleans (40% 1 RM)	Lactate: ↑ post all WODs and > after WOD 1 than after WOD 2
Durkalec-Michalski et al. (2018)	Acute	Placebo suppl. (5 men and 4 women) and sodium bicarbonate suppl. (7 men and 5 women); trained >2 years	Pre and 3′ post	Fight Gone Bad: 3 × 5′ AMRAP: 1′ wall ball (9 kg), 1′ SDHP (34 kg), 1′ box jump (50 cm), 1′ push press (34 kg), 1′ row (max cal) ICT: cycloergometer #75 w/50 w (men/women) with increase of 25 w per 1.5 min until exhaustion	Lactate and glycemia: ↑ 3′ post in both groups
Kliszczewicz et al. (2018a)	Acute	Men (*n* = 10); trained ≥3 months	Pre and post	WOD 1—For time: 30 power C&Js WOD 2−15′ AMRAP: 250 m row, 20 KB swings, 15 DB thrusters	Lactate: ↑ post both WOD
Maté-Muñoz et al. (2018)	Acute	Men (*n* = 32); trained >6 months in strength training	Pre and 3′ post	WOD 1 (Cindy): 20′ AMRAP: 5 pull-ups, 10 push-ups and 15 squats. WOD 2 (Tabata): 8 × 20″/10″ rest DUs WOD 3−5′ AMRAP power cleans (40% 1 RM)	Lactate: ↑ post all WODs and > after WOD 1 than after WOD 2, but resting lactate was > after WOD 1 than after WOD 3
Tibana et al. (2018)	Acute	Men (*n* = 9); trained >6 months	Pre, post, and 10′, 20′, and 30′ post	WOD 1 (Fran)—For time: 21-15-9 thrusters and pull-ups WOD 2 (Fight Gone Bad)−3 x AMRAP: 1′ wall ball, 1′ sumo deadlift high pull, 1′ box jump, 1′ push press, 1′ cal row, and 1′ rest	Lactate: ↑ post, 10′, 20′, and 30′ post
Ahmad et al. (2019)	Acute	Men (*n* = 17); active (hockey and football players) with experience time not described	Post 1 juice suppl. and post 2 carbohydrate-electrolyte suppl.	2 × Cindy−20′ AMRAP: pull-ups, 10 push-ups, and 15 air squats	Lactate: NS between post 1 and 2
Cadegiani et al. (2019)	Descriptive of cross-sectional cohort	Healthy men (*n* = 21) and men with overtraining (*n* = 9); trained ≥6 months; inactive men (*n* = 10)	6 months post	Training protocol not described	Lactate: < Healthy men Glycemia: NS Creatinine: NS
Coco et al. (2019)	Acute	Men (*n* = 15); professional bodybuilders	Pre, post, and 15′ post	For time (Open 15.5): 27-21-15-9 cal rows and thrusters	Lactate: ↑ post Glycemia: NS
Feito et al. (2019a)	Acute	Men (*n* = 15) and women (*n* = 14); trained >2 years	Post each round	15′ AMRAP: 250 m row, 20 KB swings (16/12 kg), and 15 dumbbell thrusters (16/9 kg)	Lactate: NS between rounds
Tibana et al. (2019b)	Acute	Men (*n* = 8); trained >6 months	pre and post each WOD	WOD 1−4′ AMRAP: 5 thrusters (60 kg) and 10 box jumps over WOD 2−4′ AMRAP: 10 power cleans (60 kg) and 20 pull-ups WOD 3−4′ AMRAP: 15 shoulder to overheads (60 kg) and 30 toes to bar WOD 4−4′ AMRAP: 20 cal rows and 40 wall balls (9 kg)	Lactate: ↑ post each WOD (in relation to pre)
Timón et al. (2019)	Acute	Men (*n* = 20); trained >1 year	pre, post, and 24 and 48 h post	WOD 1−5′ AMRAP: 1, 2, 3, 4, 5. burpees + toes to bar WOD 2—For time: 3 x 20 wall balls (9 kg), 20 power cleans (40% 1 RM)	Lactate: ↑ post-WOD 2 (in relation WOD 1) GOT and TGP: ↑ post-WOD 1 and until 24 h post-WOD 2; Gly: ↑ post-WOD 1 and 2

*WOD, workout of the day; RFT, rounds for time; AMRAP, as many rounds/repetitions as possible; NS, not significant; KB, kettlebell; DU, double under; C&J, clean and jerk; RM, maximum repetition; GOT, glutamic oxaloacetic transaminase; GPT, glutamic pyruvic transaminase*.

**Table 3:** Inflammatory responses associated with CrossFit^®^ training.

**References**	**Experimental design**	**Sample (*n*); profile experience time**	**Data collect**	**Protocol of training**	**Main findings**
Tibana et al. (2016)	Acute	Men (*n* = 9); trained >6 months	Pre, post, and 24 h and 48 h post	WOD 1−5 × 1 snatch block (2–5′ rest); 3 × 5 snatches full (90″ rest); 3 × 60″ plank hold (90″ rest); 10′ AMRAP: 30 DUs, 15 power snatches WOD 2−5 × 1 clean block (2′ rest); 5 × 1 jerk (2′ rest); 3 × 5 clean full (90″ rest); 3 × 10 strict HSPUs (2′ rest); 12′ AMRAP: 250 m row, 25 burpees touch target	IL-6: ↑ post-WOD 1 and 2 IL-10: ↑ post-WOD 1
Michnik et al. (2017)	Longitudinal	Placebo suppl. men (*n* = 9) and Green tea suppl. men (*n* = 11); well-trained with experience time not described	Pre, and 3′ and 60′ posttraining before and after suppl.	6 weeks of suppl. and CrossFit^®^ training not described	CK: ↓ post–green tea suppl. and ↑ post–placebo suppl. (in relation to before suppl.)
Durkalec-Michalski et al. (2018)	Acute	Placebo suppl. (5 men and 4 women) and sodium bicarbonate suppl. (7 men and 5 women) groups; trained >2 years	Pre and 3′ post	Fight Gone Bad: 3 × 5′ AMRAP: 1′ wall ball (9 kg), 1′ SDHP (34 kg), 1′ box jump (50 cm), 1′ push press (34 kg), 1′ row (max cal) ICT: cycloergometer at 75 W/50 W (men/women) with increase of 25 W per 1.5 min until exhaustion	CK: ↑ 3′ post in both groups
Cadegiani et al. (2019)	Descriptive of transversal cohort	Healthy men (*n* = 21) and men with overtraining (*n* = 9); trained ≥6 months; inactive men (*n* = 10)	6 months post	Training protocol not described	CK: NS difference between groups
Timón et al. (2019)	Acute	Men (*n* = 20); trained with experience time not described	Pre, post, and 24 and 48 h post	WOD 1−5′ AMRAP: 1, 2, 3, 4, 5. burpees + toes to bar WOD 2—For time: 3 x 20 wall balls (9 kg), 20 power cleans (40% 1 RM)	CK: ↑ post-WOD 1 and 2 until 24 h
Tibana et al. (2019a)	Acute	Men (*n* = 9); trained >6 months	24 h pre and 24, 48, and 72 h postcompetition	Three-day competition: WOD 1—For time in the group, *Athlete* A: Row 500 m, 30 strict HSPUs, 15 ring muscle-ups *Athlete* B: Row 1,000 m, 24 strict HSPUs, 12 ring muscle-ups *Athlete* C: Row 1,500 m, 18 strict HSPUs, 9 ring muscle-ups WOD 2—For time for each athlete: 15 fat bar hang power cleans (40 kg), 20 m overhead walking lunges (40 kg), 25 toes to bars, 10 fat bar shoulder to overhead (40 kg), 20 m overhead walking lunges (40 kg) WOD 3—For time for all athletes: 27 burpee box jump-overs, 21 legless rope climbs WOD 4—For time for each athlete: 15 m handstand walk, 6-4-2 squat snatches (60-70-85 kg), 15 m handstand walk WOD 5—For time for all athletes: 30 cal assault bike, 20 thrusters (50 kg), 40 cal assault bike, 16 thrusters (60 kg), 50 cal assault bike, 12 thrusters (70 kg), 60 cal assault bike, 8 thrusters (75 kg)	IL-10: NS CK: ↓ 72 h postcompetition

*WOD, workout of the day; AMRAP, as many rounds/repetitions as possible; Suppl., supplementation; HSPU, handstand push-up; IL-6, interleukin-6; IL-10, interleukin-10; CK, creatine kinase; SDHP, sumo deadlift high pull; ICT, incremental cycling test; W, watts; NS, not significant; RM, maximum repetitions*.

### Assessment of Risk of Bias in Included Studies

The Cochrane Collaboration's risk of bias assessment tool was used to evaluate the internal validity of the studies (Higgins et al., 2011). Selection bias (random sequence generation and allocation concealment), performance bias (blinding of participants and researchers), detection bias (blinding of outcome assessment), attrition bias (incomplete outcome data), reporting bias (selective reporting), and other biases (anything else, ideally pre-specified) were evaluated. A researcher (JGV) classified each of the factors described as low, unclear, or high risk of bias. Then a meeting was held with a second researcher (MRD) to discuss possible classification disagreements and make the final decision. In addition, a descriptive cross-sectional cohort study (Cadegiani et al., 2019) was not analyzed.

## Results

The initial search identified 623 titles in the database. First, 269 duplicate titles were excluded. After a review of the titles and abstracts of 354 articles, 28 articles (4.5%) were selected for a complete full-text review. Finally, 25 articles were selected (8 articles on hormone responses, 19 on metabolic responses and 6 on inflammatory responses). All were published between 2015 and 2019, and most were of a cross-sectional design.

The study design was described as acute (*n* = 21; pre-training and post-training or comparison between groups), longitudinal (*n* = 3; up to 6 weeks of CrossFit^®^ training) or descriptive of a cross-sectional cohort (*n* = 1; after training only).

### Sample Characteristics

The articles evaluated a total of 427 subjects. The sample sizes ranged from 8 to 40 subjects. Among the subjects, 417 were CrossFit^®^ trained or active for more than 3 months. In total, 345 subjects were men, 72 were women, and the sex of 10 participants was not specified. Most studies included men alone, 7 studies included men and women, and no studies enrolled only women.

### CrossFit^®^ Training Protocol

Different exercise protocols that are commonly prescribed in CrossFit^®^ training were found. Four articles (Murawska-Cialowicz et al., 2015; Michnik et al., 2017; Cadegiani et al., 2019; Poderoso et al., 2019) did not describe the training protocol. The studies that described the training protocols analyzed participants who performed different combinations of multiple types of exercise modalities. CV, gymnastic and weightlifting exercises): gymnastic (*n* = 2; Shaw et al., 2015; Ahmad et al., 2019), G + W (*n* = 4; Fernandez-Fernandez et al., 2015; Escobar et al., 2016, 2017; Timón et al., 2019), CV + weightlifting (*n* = 6; Perciavalle et al., 2016; Kliszczewicz et al., 2017, 2018a,b; Coco et al., 2019; Feito et al., 2019a), and CV + gymnastic + weightlifting (*n* = 9; Tibana et al., 2016, 2018, 2019a,b; Maté-Muñoz et al., 2017, 2018; Durkalec-Michalski et al., 2018; Mangine et al., 2018, 2019).

The training intensity was verified in a few studies through heart rate (HR) assessment, in which the HR remained between 85.9 and 97.4% of the maximum HR (Fernandez-Fernandez et al., 2015; Murawska-Cialowicz et al., 2015; Kliszczewicz et al., 2018a,b; Maté-Muñoz et al., 2018; Tibana et al., 2018; Ahmad et al., 2019; Feito et al., 2019a). Regarding the WOD time, 10 studies used protocols with times <10 min (Fernandez-Fernandez et al., 2015; Kliszczewicz et al., 2017, 2018a,b; Maté-Muñoz et al., 2017, 2018; Mangine et al., 2018, 2019; Tibana et al., 2018; Timón et al., 2019), 13 studies used protocols with times between 10 and 19 min (Murawska-Cialowicz et al., 2015; Shaw et al., 2015; Escobar et al., 2016, 2017; Kliszczewicz et al., 2017, 2018a,b; Maté-Muñoz et al., 2017; Durkalec-Michalski et al., 2018; Mangine et al., 2018, 2019; Tibana et al., 2018; Feito et al., 2019a), and five studies used protocols with times over 20 min (Fernandez-Fernandez et al., 2015; Maté-Muñoz et al., 2017, 2018; Mangine et al., 2018; Tibana et al., 2019b).

The frequency of training was reported in only two longitudinal studies (Murawska-Cialowicz et al., 2015; Poderoso et al., 2019). Poderoso et al. (2019) and Murawska-Cialowicz et al. (2015) adopted 5 and 2 days a week, respectively, without describing the training protocol. One study did not report weekly training frequency (Michnik et al., 2017).

### Hormonal Responses

Eight studies (Kliszczewicz et al., 2017, 2018a,b; Mangine et al., 2018, 2019; Cadegiani et al., 2019; Poderoso et al., 2019; Tibana et al., 2019b) investigated hormonal responses (see Table 1). The variables analyzed were testosterone and cortisol (Mangine et al., 2018; Cadegiani et al., 2019; Poderoso et al., 2019; Tibana et al., 2019a), GH and IGF-1 (Kliszczewicz et al., 2018a; Cadegiani et al., 2019), or adrenaline and noradrenaline (Kliszczewicz et al., 2017, 2018b; Cadegiani et al., 2019; Mangine et al., 2019).

As an acute effect, testosterone and cortisol increased after training and appeared to remain elevated 30 and 60 min after some training protocols (Mangine et al., 2018). Testosterone and cortisol returned to their initial values 48 h after training (Tibana et al., 2019a). In the only longitudinal study (Poderoso et al., 2019), testosterone was higher after the 6th month, and cortisol was lower after the 4th month. Comparing different groups (healthy men, men with overtraining, and inactive men), Cadegiani et al. (2019) showed that healthy men presented a higher testosterone level than men with overtraining, while cortisol was not different among them.

In the study by Kliszczewicz et al. (2018a), GH was higher 1 h after training involving CV and weightlifting exercises compared with weightlifting exercises only. Like testosterone, when compared between different groups, GH was higher in healthy men than in inactive men (Cadegiani et al., 2019). IGF-1 showed no differences soon after WODs or between different groups.

Adrenaline and noradrenaline were higher soon after training regardless of the WOD (Kliszczewicz et al., 2017, 2018b; Mangine et al., 2019). After 6 months of training, the resting adrenaline was not different, while noradrenaline was lower with overtraining (Cadegiani et al., 2019).

### Metabolic Responses

Nineteen studies (Fernandez-Fernandez et al., 2015; Murawska-Cialowicz et al., 2015; Shaw et al., 2015; Escobar et al., 2016, 2017; Perciavalle et al., 2016; Tibana et al., 2016, 2018, 2019a; Kliszczewicz et al., 2017, 2018a; Maté-Muñoz et al., 2017, 2018; Durkalec-Michalski et al., 2018; Ahmad et al., 2019; Cadegiani et al., 2019; Coco et al., 2019; Feito et al., 2019a; Timón et al., 2019) investigated metabolic responses (see Table 2). The metabolic variables analyzed were lactate (Fernandez-Fernandez et al., 2015; Murawska-Cialowicz et al., 2015; Shaw et al., 2015; Escobar et al., 2016, 2017; Perciavalle et al., 2016; Tibana et al., 2016, 2018, 2019b; Maté-Muñoz et al., 2017, 2018; Durkalec-Michalski et al., 2018; Kliszczewicz et al., 2018a; Ahmad et al., 2019; Cadegiani et al., 2019; Coco et al., 2019; Feito et al., 2019a; Timón et al., 2019), glycemia (Shaw et al., 2015; Perciavalle et al., 2016; Tibana et al., 2016; Kliszczewicz et al., 2017; Durkalec-Michalski et al., 2018; Cadegiani et al., 2019; Coco et al., 2019; Timón et al., 2019), cholesterol (Shaw et al., 2015), creatinine (Cadegiani et al., 2019), GOT and GPT (Timón et al., 2019).

Most studies investigated lactate responses (17 studies). There was a consensus that lactate levels are high immediately after a CrossFit^®^ training session. This increase seems to occur at the beginning of the training and persists for up to 30 min after the session (Tibana et al., 2018). Some studies presented differences between the different WODs (Tibana et al., 2016; Maté-Muñoz et al., 2017, 2018; Timón et al., 2019), and some found no differences (Fernandez-Fernandez et al., 2015; Durkalec-Michalski et al., 2018; Tibana et al., 2018, 2019b). Chronically, Murawska-Cialowicz et al. (2015) reported non-significant changes in lactate values after 3 months of CrossFit^®^ training. Cadegiani et al. (2019), when evaluating healthy athletes and athletes with overtraining syndrome, found that healthy athletes had lower blood lactate levels than those with overtraining who followed an undescribed training protocol. The pre-WOD lactate values were ~2.9 mmol·L^−1^ for all studies. However, blood lactate responses after the protocols were similar regardless of the WOD time. The values presented were 10.15–18.38 mmol·L^−1^ for protocols with a time <10 min (Maté-Muñoz et al., 2017, 2018; Kliszczewicz et al., 2018a; Tibana et al., 2018; Timón et al., 2019), 5.95–16.56 mmol·L^−1^ for protocols with a time ranging from 10 to 19 min (Maté-Muñoz et al., 2017, 2018; Kliszczewicz et al., 2018a; Tibana et al., 2018; Timón et al., 2019) and 11.79–17.43 mmol·L^−1^ for protocols ≥20 min (Fernandez-Fernandez et al., 2015; Maté-Muñoz et al., 2017, 2018; Ahmad et al., 2019; Tibana et al., 2019b). The values presented were 6.34–14.0 mmol·L^−1^ for studies that did not report the duration of the protocols (Fernandez-Fernandez et al., 2015; Perciavalle et al., 2016; Tibana et al., 2016; Coco et al., 2019).

Glycemia was often investigated (8 studies). Similar to lactate, several studies showed an increase after independent WOD (Tibana et al., 2016; Kliszczewicz et al., 2017; Durkalec-Michalski et al., 2018; Timón et al., 2019), while other studies did not show changes in glycemic levels after training (Shaw et al., 2015; Perciavalle et al., 2016; Coco et al., 2019). Only the study by Tibana et al. (2016) showed that blood glucose changed after training as a function of the WOD type. Blood glucose did not change between healthy and overtraining syndrome athletes in an undescribed training protocol (Cadegiani et al., 2019).

Cholesterol, creatinine, GOT and GPT were each investigated in only one study. Cholesterol showed no differences after training (Shaw et al., 2015), and creatinine did not change after 6 months of CrossFit^®^ training (Cadegiani et al., 2019). Timón et al. (2019) were the only researchers to evaluate GOT and GPT responses after training, and their results showed significant increases values independent of the WOD.

### Inflammatory Responses

Six studies (Tibana et al., 2016, 2019a; Michnik et al., 2017; Durkalec-Michalski et al., 2018; Cadegiani et al., 2019; Timón et al., 2019) investigated inflammatory responses (see Table 2). Biomarkers of muscle damage analyzed were CK (Michnik et al., 2017; Durkalec-Michalski et al., 2018; Cadegiani et al., 2019; Tibana et al., 2019a; Timón et al., 2019), IL-6 and IL-10 (Tibana et al., 2016, 2019a).

CK seemed to increase after training (Durkalec-Michalski et al., 2018; Timón et al., 2019) or to decrease within 72 h after training (Tibana et al., 2019a). Michnik et al. (2017) found that after ingestion of green tea, CK decreased after training. As a chronic effect, there were no differences after 6 months of training (Cadegiani et al., 2019).

IL-6 increased after WOD-independent training, while IL-10 increased as a function of WOD characteristics (Tibana et al., 2016). Comparing five different WODs, IL-10 showed no differences after training (Tibana et al., 2019a).

### Risk of Bias in Included Studies

**Figure 2:**
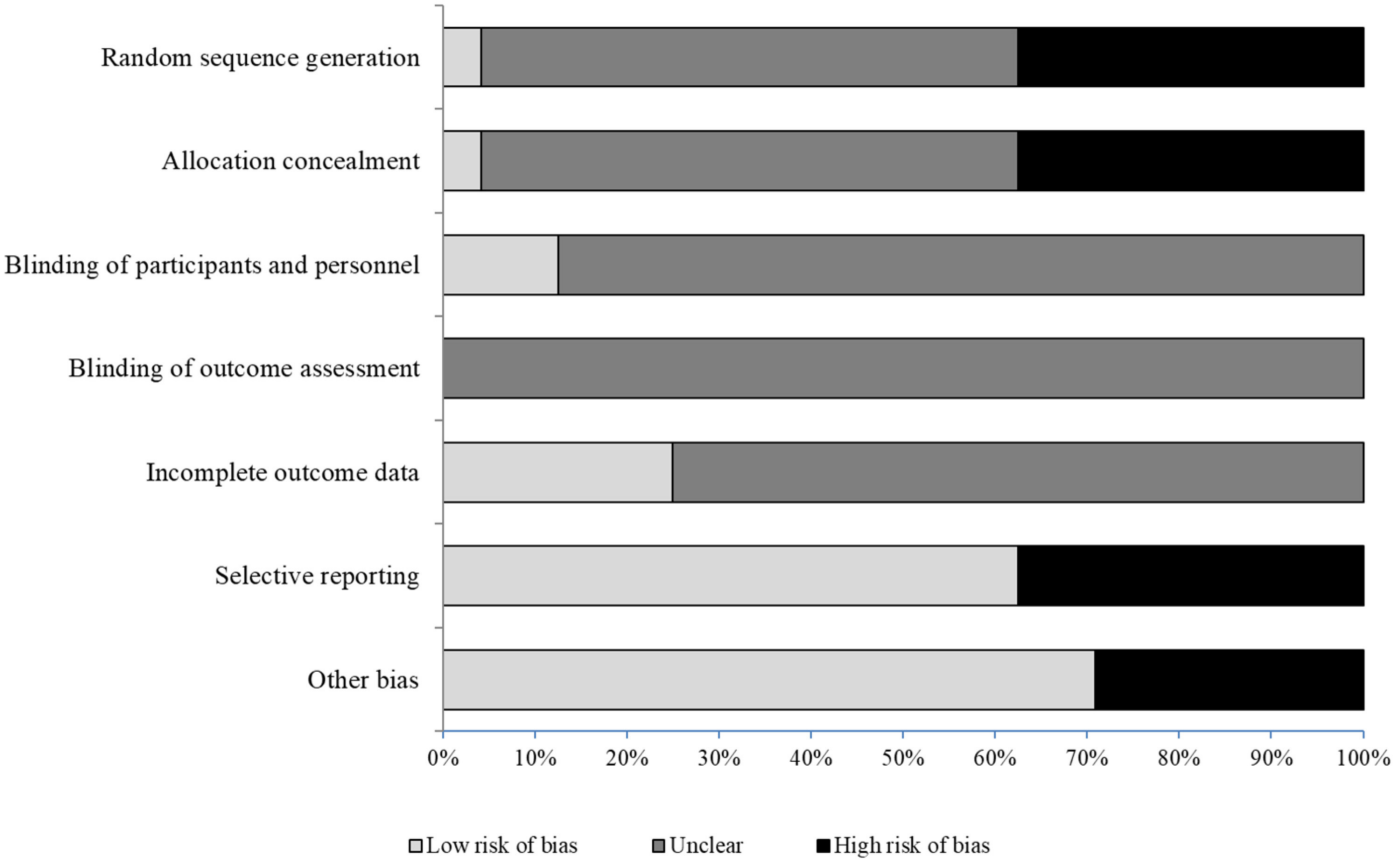
Cochrane risk of bias assessment.

Table 4 summarizes the results of the methodological quality assessment across all included studies and Figure 2 shows the percentage distribution of quality. Procedures for a random sequence generation and allocation concealment were unclear in 14 of 24 studies. A low risk of bias was found in three trials regarding blinding of participants/personnel. Six studies showed complete outcome data. Seven out of 24 studies showed high risk of “other bias.” These studies did not describe the training protocol or concealed participants' data.

**Table 4:** Risk of bias assessment.

**References**	**Random sequence generation**	**Allocation concealment**	**Blinding of participants and personnel**	**Blinding of outcome assessment**	**Incomplete outcome data**	**Selective reporting**	**Other bias**
Kliszczewicz et al. (2017)	Unclear risk	Unclear risk	Unclear risk	Unclear risk	Low risk	High risk	Low risk
Kliszczewicz et al. (2018a)	Unclear risk	Unclear risk	Unclear risk	Unclear risk	Low risk	High risk	Low risk
Kliszczewicz et al. (2018b)	Unclear risk	Unclear risk	Unclear risk	Unclear risk	Low risk	High risk	Low risk
Mangine et al. (2018)	High risk	High risk	Unclear risk	Unclear risk	Low risk	High risk	Low risk
Mangine et al. (2019)	High risk	High risk	Unclear risk	Unclear risk	Low risk	High risk	Low risk
Poderoso et al. (2019)	High risk	High risk	Unclear risk	Unclear risk	Unclear risk	Low risk	High risk (training protocol not described)
Tibana et al. (2019a)	Unclear risk	Unclear risk	Unclear risk	Unclear risk	Unclear risk	Low risk	Low risk
Fernandez-Fernandez et al. (2015)	Unclear risk	Unclear risk	Unclear risk	Unclear risk	Unclear risk	Low risk	High risk (gender not described)
Murawska-Cialowicz et al. (2015)	High risk	High risk	Unclear risk	Unclear risk	Unclear risk	Low risk	High risk (training protocol not described)
Shaw et al. (2015)	High risk	High risk	Unclear risk	Unclear risk	Unclear risk	Low risk	Low risk
Escobar et al. (2016)	Unclear risk	Unclear risk	Low risk	Unclear risk	Unclear risk	Low risk	Low risk
Perciavalle et al. (2016)	High risk	High risk	Unclear risk	Unclear risk	Unclear risk	High risk	High risk (age of participants not described)
Tibana et al. (2016)	Unclear risk	Unclear risk	Unclear risk	Unclear risk	Unclear risk	Low risk	Low risk
Escobar et al. (2017)	High risk	High risk	Unclear risk	Unclear risk	Unclear risk	Low risk	Low risk
Maté-Muñoz et al. (2017)	Unclear risk	Unclear risk	Unclear risk	Unclear risk	Unclear risk	High risk	Low risk
Durkalec-Michalski et al. (2018)	Low risk	Low risk	Low risk	Unclear risk	Low risk	Low risk	Low risk
Maté-Muñoz et al. (2018)	Unclear risk	Unclear risk	Unclear risk	Unclear risk	Unclear risk	High risk	Low risk
Tibana et al. (2018)	Unclear risk	Unclear risk	Unclear risk	Unclear risk	Unclear risk	Low risk	Low risk
Ahmad et al. (2019)	Unclear risk	Unclear risk	Low risk	Unclear risk	Unclear risk	Low risk	High risk (experience time not described)
Coco et al. (2019)	High risk	High risk	Unclear risk	Unclear risk	Unclear risk	High risk	High risk (age of participants not described)
Feito et al. (2019a)	High risk	High risk	Unclear risk	Unclear risk	Unclear risk	Low risk	Low risk
Timón et al. (2019)	Unclear risk	Unclear risk	Unclear risk	Unclear risk	Unclear risk	Low risk	Low risk
Tibana et al. (2019b)	Unclear risk	Unclear risk	Unclear risk	Unclear risk	Unclear risk	Low risk	Low risk
Michnik et al. (2017)	Unclear risk	Unclear risk	Unclear risk	Unclear risk	Unclear risk	Low risk	High risk (experience time not described; training protocol not described)

## Discussion

The purpose of this review was to systematically examine the existing literature on characterization of hormonal, metabolic, and inflammatory responses associated with CrossFit^®^ training. The results of this systematic review showed that there are still few studies for each observed variable. Of the 25 studies analyzed, all had different training protocols regarding the training stimulus administered. This methodological difference was found because CrossFit^®^ training is constantly varied and consists of a combination of different exercise elements: CV, gymnastic and weightlifting exercises (Glassman, 2017). However, the hormonal, metabolic, and inflammatory changes induced by CrossFit^®^ training seem to be related to the training variables, and the protocols with more volume and intensity provided greater biochemical responses. In addition, psychological factors, such as pre-competitive anxiety, can alter the physiological status of athletes (Mangine et al., 2018).

### Hormonal Responses

The studies demonstrated that increases in testosterone and cortisol levels occurred after WODs, with longer recovery intervals (Mangine et al., 2018; Tibana et al., 2019a). However, testosterone and cortisol levels decreased after 48 h of training (Tibana et al., 2019a). Acute increases in testosterone may have different explanations: (a) transient increases in strength during training (Hooper et al., 2017); (b) repetitions with maximum effort, which generates additional overload (Ahtiainen et al., 2003). On the other hand, the elevation in cortisol levels, can be explained by psychological factors, as observed in different types of sport athletes (Casto and Edwards, 2016). The perception of the difficulty of the WODs and the stimulus to perform a task for time or AMRAP are other factors that can increase the psychological stress of CrossFit^®^ training practitioners and create an environment conducive to an increase in cortisol (Mangine et al., 2019).

Some limitations were observed in the study by Mangine et al. (2018), who evaluated acute testosterone and cortisol changes. In a small sample of five men and five women, one woman was on an oral contraceptive (medroxyprogesterone). Testosterone and cortisol responses were similar to those in women's soccer, volleyball, and softball athletes (Edwards and O'Neal, 2009) and hockey players (Crewther et al., 2015). Further studies are needed to verify that such responses are consistent with women who participate in CrossFit^®^ training.

Chronically, testosterone levels in men rose after 6 months of CrossFit^®^ training, while cortisol levels decreased (Poderoso et al., 2019). The high-volume and high-intensity protocols with short rest intervals between sets tended to lead to greater hormonal changes (Kraemer and Ratamess, 2005). Previous studies have reported a significant increase in testosterone levels after high-intensity exercises in runners and master athletes of various modalities (Herbert et al., 2017; Nuuttila et al., 2017). Women did not present significant chronic testosterone and cortisol changes (Poderoso et al., 2019). It can be expected that women do not undergo major adaptations in testosterone levels due to their low responsiveness (Kraemer et al., 1998).

The training dose required to cause hormonal changes is difficult to determine. Notably, training with a greater volume promoted increases in GH concentrations (Kliszczewicz et al., 2018a) but did not alter catecholamine levels (Kliszczewicz et al., 2018b). Regardless of time, different intensity of exercises increased testosterone levels, while training with longer duration increased the concentration of cortisol (Mangine et al., 2018). It seems that isolated exercises become competitors when combined in a single training session. For example, when subjected to isolated resistance training, runners showed a decrease in testosterone and an increase in cortisol (Anderson et al., 2016). Performing endurance exercises before strength training can increase the cortisol and lactate concentrations in the blood of recreational strength training practitioners (Jones et al., 2016). We suggest that the performance of WODs with a predominance of CV exercises before weightlifting exercises results in unfavorable hormonal responses.

On the other hand, increases in testosterone levels may be closely related to the health of the participants. In a descriptive cross-sectional study, Cadegiani et al. (2019) showed that healthy CrossFit^®^ training practitioners have higher levels of testosterone and GH and lower levels of noradrenaline than practitioners with overtraining syndrome. The higher levels of noradrenaline in practitioners with overtraining syndrome may be a compensatory attempt to maintain performance during exercise due to reduced conversion of catecholamines to metanephrines (Cadegiani et al., 2019). The fact that CrossFit^®^ training has a high metabolic component may explain the increases in GH levels (Kliszczewicz et al., 2018a). Thus, increases in blood lactate can enhance GH levels due to their relationship, especially when the modality is not interval training but rather a more continuous exercise (Goto et al., 2005). Another factor that can influence increases in GH levels is the increased release induced by noradrenaline in the hypothalamus, which inhibits somatostatin, the main inhibitor of GH release in the pituitary gland (Kliszczewicz et al., 2018b). Unlike GH, IGF-1 does not increase significantly after a high-intensity functional training session (Kliszczewicz et al., 2018a). The lack of response from IGF-1 may be partially related to the regulatory role of GH in IGF-1 levels, affecting its post-exercise response (Duan and Xu, 2005). On the other hand, Borst et al. (2001) observed significant increases in IGF-1 in response to longitudinal training of 13 weeks.

Catecholamines showed acute elevations after WODs (Kliszczewicz et al., 2017, 2018b; Mangine et al., 2019). Generally, with high-intensity training, there is an increase in the sympathetic response and a concomitant need to increase hemodynamic parameters (Mangine et al., 2019). However, it was observed that 3 h after training, levels decreased compared to pre-exercise levels (Kliszczewicz et al., 2017). Kraemer et al. (2015) reported that catecholamine levels increased during resistance, aerobic, and combined training as the intensity increased. After training (5 and 15 min), the levels appeared to return to baseline in response to decreased physical activity and increased parasympathetic activity. Autonomic control attempts to re-establish homeostasis, which is likely the reason why noradrenaline decreased below the baseline in the Mangine et al. (2019) study.

When comparing the chronic hormonal responses of CrossFit^®^ practitioners with other sport athletes, the responses may differ. From this perspective, Arruda et al. (2015) observed that young soccer players experienced a drop in testosterone levels as the season progressed, which appears to be different in men who practice CrossFit^®^ training (Poderoso et al., 2019). CrossFit training is characterized by the combination and variation of different elements in the same session, i.e., CV, gymnastic and weightlifting. Thus, further studies are needed to compare hormonal responses in different sports.

### Metabolic Responses

The main metabolic marker evaluated in CrossFit^®^ training studies was blood lactate. An acute increased lactate concentration response was observed after training sessions (Fernandez-Fernandez et al., 2015; Murawska-Cialowicz et al., 2015; Shaw et al., 2015; Escobar et al., 2016; Perciavalle et al., 2016; Tibana et al., 2016, 2018, 2019a; Maté-Muñoz et al., 2017, 2018; Durkalec-Michalski et al., 2018; Kliszczewicz et al., 2018a; Coco et al., 2019; Timón et al., 2019) and remained elevated for up to 30 min after the end of the session (Tibana et al., 2018). In CrossFit^®^ training, WODs generally do not have a standard break time, i.e., as the training is “for time” or AMRAP, the intervals are self-selected according to the suitability of the participants. Therefore, this characteristic can keep lactate elevated for a longer duration after the session (Goto et al., 2005).

As for chronic metabolic responses, the lack of change in lactate response may be the result of the intensity utilized for each WOD. It must also be considered that pre-training lactate was not registered (Murawska-Cialowicz et al., 2015). Thus, at present, chronic metabolic responses in CrossFit^®^ training practitioners are inconclusive.

Blood glucose was another variable observed. According to Timón et al. (2019) and Kliszczewicz et al. (2017), the glycemic rate rises after a CrossFit^®^ training session due to increased catecholamines. The increase in the glycemic rate in response to a training session is due to the need for greater utilization of glucose to meet the energy required for the sport, which has the particular characteristic of always being performed at high intensity (Glassman, 2017). However, other studies did not observe significant acute changes in blood glucose (Shaw et al., 2015; Perciavalle et al., 2016; Coco et al., 2019), and one study found that the results depend on the practitioner's performance status (Cadegiani et al., 2019). Only one study investigated chronic blood glucose responses with 8 weeks of HIFT and aerobic/resistance training (Feito et al., 2019b), reporting no significant differences in blood glucose responses between groups.

Cholesterol response does not appear to be affected by a CrossFit^®^ training session (Shaw et al., 2015); apparently, the session time was insufficient (10-min AMRAP) to change total cholesterol responses. Hepatic transaminases (GOT and GPT) were analyzed to assess liver overload during exertion. In both WODs, there was a significant increase after exercise (Timón et al., 2019), and these levels remained high at 24 h only after WOD 2, which made the results curious given the higher intensity of WOD-2 compared to WOD-1. One study even claimed that intensive muscle exercise (e.g., weightlifting) can increase hepatocellular damage for up to 7 days after exercise (Pettersson et al., 2008). The accelerated metabolic demands of muscle exercise cannot be met without a significant liver response (Trefts et al., 2015).

### Inflammatory Responses

Acute muscle damage responses through CK were investigated in three studies (Durkalec-Michalski et al., 2018; Tibana et al., 2019b; Timón et al., 2019). Durkalec-Michalski et al. (2018) found that CK levels increased significantly after benchmark “Fight Gone Bad” and incremental cycle ergometer testing. However, this study examined the effects of sodium bicarbonate intake on CrossFit^®^ training performance and aerobic capacity. Although the primary objective of the study was to examine the effectiveness of a sodium bicarbonate supplementation protocol, the CK response was similar, regardless of the supplement, increasing after the WOD. Another study found an increase in CK level after different WODs, which continued for up to 24 h (Timón et al., 2019). These results are consistent with those of strength training, which were shown to increase CK levels in 58 individuals after performing 4 min of high-intensity interval resistance training. The high-intensity interval resistance training session consisted of eight sets of squats performed with the fastest speed and highest number of repetitions achievable for 20 s, with 10 s of rest between sets (Spada et al., 2018). Moreover, it seems that CK decreases after 72 h (Tibana et al., 2019a), for which we speculate that after 3 successive days of competition, athletes adapt to high-intensity stimuli. In addition, certain factors, such as level of training, muscle groups involved, and sex, can influence CK levels more than differences in exercise volume or type (Koch et al., 2014).

In addition to being an indicator of muscle damage, CK levels have been shown to be high in people with rhabdomyolysis (Honda et al., 2017; Meyer et al., 2017; Nadaf et al., 2018). The incidence of exertional rhabdomyolysis remains unclear, probably because exercise varies in intensity and individual tolerance. However, it remains controversial whether the intensity of the exercise was consistent with the markedly elevated levels of serum CK (Honda et al., 2017). Chen et al. (2019) found with resistance training, that CK levels are higher when training is on consecutive days. Thus, subjecting CrossFit^®^ training practitioners to a higher volume and intensity of training for successive days may expose the practitioner to the risk of damage associated with muscle cell necrosis.

IL-6 and IL-10 were evaluated in two studies (Tibana et al., 2016, 2019a). Tibana et al. (2016) showed that after training the IL-6 and IL-10 levels increased in relation to baseline levels. IL-6 was considered an multifunctional cytokine produced partly by leukocytes as an inflammatory response to exercise (Suzuki et al., 2020). In addition, IL-6 release may be affected by carbohydrate bioavailability (Nieman et al., 2003), which has been suggested to be relevant to skeletal muscle energy supply (Petersen and Pedersen, 2005). An increase in IL-6 may trigger an increase in IL-10 (Petersen and Pedersen, 2005). Recently, Tibana et al. (2019a) found that IL-10 levels did not change significantly 24 h after 3 consecutive days of CrossFit^®^ competition. In contrast, Heavens et al. (2014) showed that after an adapted protocol of functional fitness, muscle damage-related (CK) and inflammatory (IL-6) biomarkers increased significantly, with the peak being found 24 h after the session. Perhaps the sample differences explain the conflicting results; Tibana et al. (2019a) used men experienced in the training, while Heavens et al. (2014) studied inexperienced individuals. The body adapts so that a single training session protects against muscle damage resulting from subsequent sessions, i.e., the repeated bout effect (Hyldahl et al., 2017).

## Conclusion

The present review demonstrates the potentially significant effect of CrossFit^®^ training on hormonal, metabolic and inflammatory factors. However, studies evaluating such aspects have a low level of evidence and reliability due to methodological limitations and biases that hinder the convergence of results. Apparently, hormonal, metabolic, and inflammatory stress marker levels increase after CrossFit^®^ training, regardless of the protocol used. However, a greater volume and intensity of workouts accentuate the responses. Some parameters are inconclusive, such as blood glucose and IL-6 and IL-10 levels, due to different results and the small number of studies. Thus, this review sheds light on specific knowledge gaps that should be further investigated. Nevertheless, the results are of paramount importance for improving understanding of the effects of CrossFit^®^ training and serve as a basis for prescribing future exercise protocols.

## Data Availability Statement

All relevant data is contained within the article. Further inquiries can be directed to the corresponding author.

## Author Contributions

NJ structured and designed the research. NJ and MD carried out the review of studies. JGV and MD realized risk of bias. NJ, JN, JGV, MD, and JV contributed to the conception and writing of the article, reviewing, and editing the manuscript. DB corrected the final version and English grammar. All authors approved the final version.

## Conflict of Interest

The authors declare that the research was conducted in the absence of any commercial or financial relationships that could be construed as a potential conflict of interest.

## Abbreviations

GH: Growth hormone
IGF-1: Insulin-like growth factor 1
GOT: Glutamic oxalacetic transaminase
GPT: Glutamic pyruvic transaminase
IL-6: Interleukin 6
IL-10: Interleukin 10
CK: Creatine Kinase
CV: Cardiovascular.
